# The Effects of Mindfulness Meditation on Mechanisms of Attentional Control in Young and Older Adults: A Preregistered Eye Tracking Study

**DOI:** 10.1523/ENEURO.0356-23.2025

**Published:** 2025-07-22

**Authors:** Andy Jeesu Kim, Keran Chen, Ying Tian, Mara Mather

**Affiliations:** School of Gerontology, University of Southern California, Los Angeles, California 90089

**Keywords:** aging, attentional control, locus ceruleus, mindfulness meditation, visual search

## Abstract

Neuroimaging data reveal that a functional locus ceruleus-noradrenaline (LC-NA) system is critical in maintaining cognitive performance during aging. However, older adults show reduced LC integrity and altered functional connectivity, demonstrating both structural declines and dysfunction. The LC-NA system mediates mechanisms of attention processing and eye tracking studies have shown that older adults are slower and more distractible compared with young adults in visual search tasks. Prior studies have shown that mindfulness meditation modulates LC noradrenergic activity, increases gray matter volume in the brainstem, and improves attentional control. Thus, in a preregistered longitudinal study, we investigated whether 30 d of guided mindfulness meditation using a mobile application improved attentional control measured with eye movements. We hypothesized that older adults would show greater benefits from the mindfulness intervention compared with young adults. In two oculomotor search tasks, we identified that guided mindfulness practice improved saccadic reaction times, but that other longitudinal benefits in goal-directed attentional control or distractibility by task-irrelevant salient stimuli may be from repeated practice. Furthermore, we did not find evidence for age differences in response to the mindfulness intervention between young, middle-aged, and older adults, nor among scores on mindfulness questionnaires. Our findings show that short-term mindfulness practice can modulate cognition, specifically with the speed of overt orienting of attention that may not be observable in self-report measures. This study is the first to show the utility of mindfulness on cognition using a highly reliable measure in eye movements and suggests future longer-term intervention studies may be warranted.

## Significance Statement

In a preregistered study, we investigated whether 30 d of guided mindfulness meditation can improve mechanisms of attentional control measured with eye tracking. We identified that mindfulness improved saccadic reaction times but found that the benefits did not differentially affect young, middle-aged, or older adults. Our findings reveal that short-term mindfulness practice can improve attention processing in ways that may not be observable in self-report questionnaires.

## Introduction

The locus ceruleus (LC) is the brain's primary source of noradrenaline and a functional locus ceruleus-noradrenaline (LC-NE) system is fundamental for healthy aging (Wilson et al., 2013; Takahashi et al., 2015; Clewett et al., 2016; Mather and Harley, 2016). The earliest signs of Alzheimer's tau pathology disease progression are found in the LC (Braak et al., 2011; Mravec et al., 2014; Braak and Del Tredici, 2015), and postmortem investigations of patients with neurodegenerative pathologies have found that LC cell death and the spread of neurofibrillary tangles are correlated with cognitive impairments (Matthews et al., 2002; Nelson et al., 2012; Theofilas et al., 2017). In human neuroimaging studies, researchers have taken advantage of the different magnetic properties in LC noradrenaline neurons to quantify LC structure (Liu et al., 2017; Betts et al., 2019) and found reduced LC MRI contrast in older adults is associated with poorer cognitive functioning (Clewett et al., 2016; Hämmerer et al., 2018; Dahl et al., 2019, 2022, 2023; Liu et al., 2020; Cassidy et al., 2022) and increased risk for mild cognitive impairment and neurodegenerative diseases (Sommerauer et al., 2018; Elman et al., 2021). Furthermore, older adults show altered functional connectivity in frontoparietal regions with the LC (Zhang et al., 2016; Lee et al., 2018), and decreased structural LC MRI contrast in older adults is associated with reduced structural thickness in frontoparietal regions (Bachman et al., 2021) indicating noradrenaline dysfunction in the aging LC is associated with changes in cortical attention networks. Tonic noradrenergic activity has been hypothesized to increase in the older brain as a compensatory mechanism (Elman et al., 2017; Weinshenker, 2018; Gannon and Wang, 2019; Mather, 2020). However, this noradrenergic hyperactivity hypothesis has not been confirmed.

The LC has numerous projections to the prefrontal cortex that facilitate executive functions including attention (Aston-Jones et al., 1999, 2000; Bouret and Sara, 2004; McGaughy et al., 2008; Reynaud et al., 2019). Optogenetic stimulation of the LC improves visual attention performance in rhesus monkeys (Ghosh and Maunsell, 2024) and modulates goal-directed attention in rodents (Bari et al., 2020). Conversely, pharmacologically inhibiting noradrenaline secretion impairs attentional control in multiple tasks (Chamberlain and Robbins, 2013). These converging findings demonstrate that LC noradrenaline activity directly modulates attention selectivity. In humans, noradrenaline facilitates functional switching between the dorsal and ventral networks of attention (Bouret and Sara, 2005; Corbetta et al., 2008). Furthermore, the firing frequency of LC neurons can be modulated by changes in arousal states (Aston-Jones and Cohen, 2005; Valentino and Van Bockstaele, 2008; Chandler, 2016), and these differences in noradrenaline discharge rates differentially facilitate cognitive behavior including attention performance (Aston-Jones et al., 1999; Sara and Bouret, 2012). Interestingly, young adults demonstrate a benefit in attentional selectivity in conjunction with increased frontoparietal network activation under elevated arousal, but older adults failed to show these improvements (Lee et al., 2018). Older adults also show deficiencies in multiple mechanisms of attentional control including goal-directed target selection, reactive disengagement of task-irrelevant stimuli, and delayed attention processing speeds (Kim et al., 2024b). Based on these findings, we hypothesize that older adults exhibit tonic noradrenergic hyperactivity and that this altered state is facilitating suboptimal attentional control.

Meditation is a popular mental practice that reduces arousal and stress and improves mental and physical health (Goyal et al., 2014). In particular, mindfulness meditation focuses on the present body and is aimed to improve self-regulatory processes such as attentional control (Jha et al., 2007; Sumantry and Stewart, 2021; Mirabito and Verhaeghen, 2023). Prior research has found that mindfulness-based techniques such as integrative body-mind training (IBMT) and mindfulness-based stress reduction (MBSR) can improve alerting and orienting (Chiesa et al., 2011) by enhancing neural activity in the anterior cingulate cortex, prefrontal cortex, and striatum (Tang et al., 2015). In addition, pretest-training-posttest mindfulness meditation intervention protocols affect the structure and function of neural networks modulating executive functions (Gallant, 2016). With regard to the LC, meditation has been shown to modulate noradrenergic activity via respiratory coupling (Craigmyle, 2013; Melnychuk et al., 2018) and to increase gray matter concentration in the brainstem and the locus ceruleus (Vestergaard-Poulsen et al., 2009; Singleton et al., 2014; Yuan et al., 2020), suggesting a potential for mindfulness meditation interventions to restore disrupted neural connections of the LC.

We hypothesize that noradrenergic dysfunction in older adults is characterized by elevated tonic discharge and contributing to suboptimal attentional control. In this study, we implemented a 30 d mindfulness meditation intervention to reduce arousal, lower tonic noradrenergic activity, and thus recover disrupted mechanisms of attentional control. We predicted that the intervention would improve mechanisms of attentional control compared with the control group and that older adults would show significantly more improvements compared with young adults.

## Material and Methods

### Power analyses and participants

To our knowledge, there are no prior investigations into the effects of mindfulness meditation intervention on attentional control measured through eye movements. However, Champion et al. (2018) used a similar mindfulness meditation intervention protocol as proposed in this study (Headspace application usage for 4 weeks) and found within-group effect sizes for participants who completed the mindfulness intervention to be *d* = 0.65, 0.83, and 0.79 for self-reported psychosocial outcomes (Champion et al., 2018). Furthermore, meta-analyses examining effects of meditation on attention outcomes have found a range of effect sizes including *d* = 0.630 (Eberth and Sedlmeier, 2012), *d* = 0.580 (Sedlmeier et al., 2012), and three-level estimate of Hedge's *g* = 0.171 (Sumantry and Stewart, 2021). Based on the effect sizes for our mindfulness meditation intervention protocol and prior effects of meditation on attention outcomes, we elected to conservatively power our study to be able to detect small-to-medium effect sizes (*f* = 0.15; Cohen, 2016) in our planned repeated-measures ANOVA analyses investigating age differences in the effects of the mindfulness meditation intervention on our measures of attentional control. In a similar oculomotor visual search task (Anderson and Kim, 2019), oculomotor capture by a high-value task-irrelevant distractor showed high test–retest reliability over two visits (*r* = 0.798), which was also a primary measure of interest in this study. Using G*Power 3.1, we computed that a sample size of 26 participants is required to observe within–between interaction effects at power (1-*β*) = 0.9 and *α* = 0.05 (number of groups = 2, number of measurements = 6). We planned to recruit older adults aged 50–80 years old. However, given this wide age range and the likelihood of increased locus ceruleus dysfunction and Alzheimer's diseases pathology with age, we planned to recruit an equal number of older adults who are aged 50–65 and 65–80 years old to conduct exploratory analyses between older adult groups. Regarding the primary measure of interest, a sample size of 20 participants is required to observe within–between interaction effects at power (1-*β*) = 0.8.

Based on our power analysis and to be sufficiently powered for exploratory analyses of differences within the two older cohorts, we proposed to recruit at least 26 young adults (YA), 20 middle-aged adults aged 50–65 years old (mA), and 20 older adults aged 65–80 years old (OA) to complete the entire experiment. Half of the participants in each age group were randomly assigned to Groups A or B (see below for details). Data from participants who dropped out from the study after the second but before the third lab visit were included in the first set of analyses comparing Visit 1 and 2 performances across counterbalancing and age groups, but not in the analyses comparing post-meditation visits across age groups (*n* = 2). Young adults between the ages of 18–30 inclusive were recruited from the University subject pool for course credit and monetary compensation. Older adults aged 50–80 inclusive were recruited from the local communities for monetary compensation. During the initial in-lab visit, older adults were screened for cognitive dysfunction and functional health using the Montreal Cognitive Assessment (MoCA; Hobson, 2015). All study protocols were approved by the University Review Board, and written informed consent was obtained from all participants prior to participation. Our final sample size comprised 28 young (mean age = 20.8, SE = 0.6; 24 female, 4 male), 20 middle-aged (mean age = 60.4, SE = 1.0; 14 female, 6 male), and 21 older adults (mean age = 74.4, SE = 0.9; 13 female, 8 male).

### Experiment procedure

**Figure 1.**
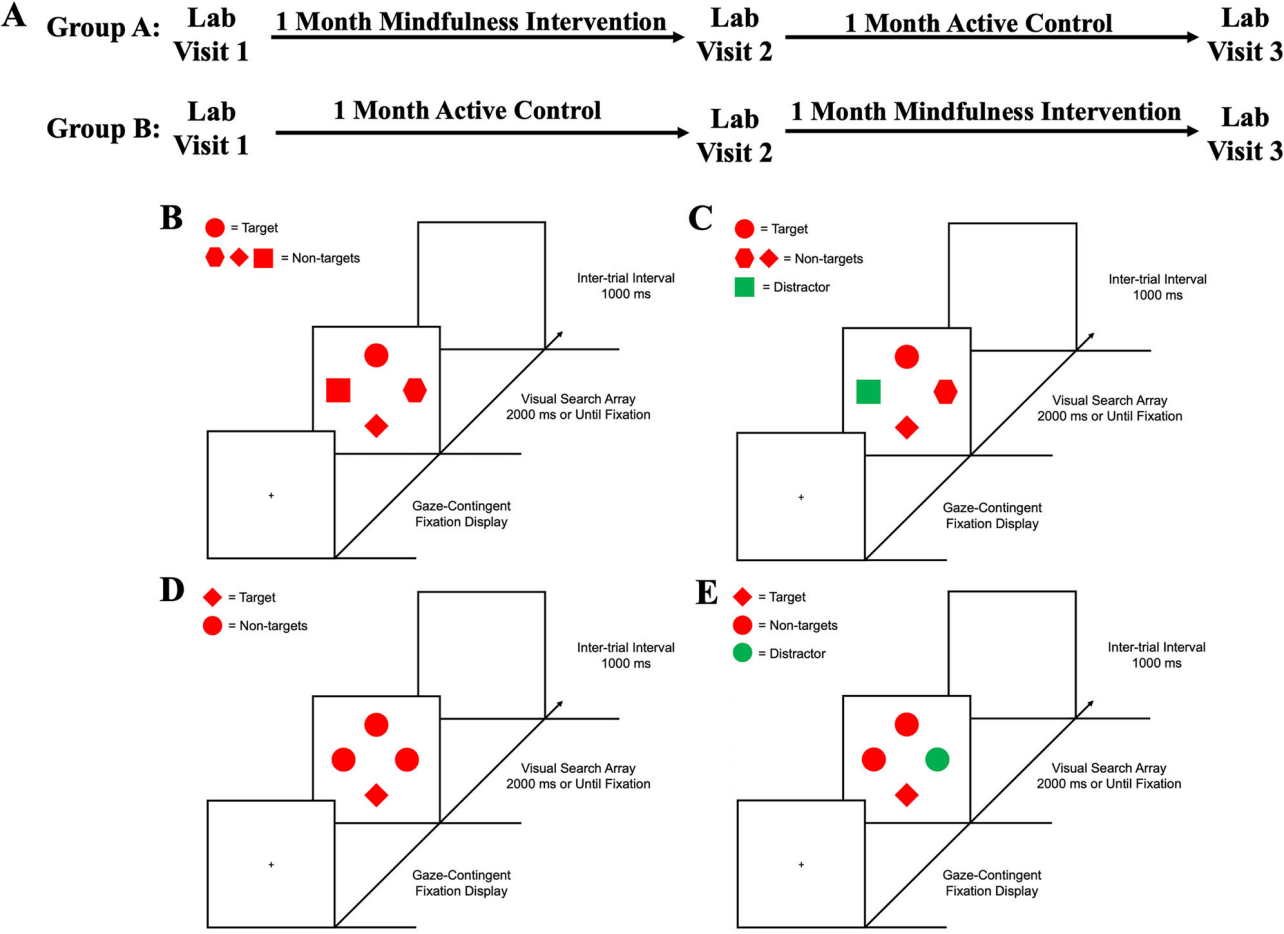
Experiment procedure and visual search tasks. ***A***, Participants were divided into two groups and completed both visual search tasks during each lab visit. The first task (feature search) included four different shapes on each trial and measured proactive inhibition of the distractor in (***B***) distractor-absent and (***C***) distractor-present trials. The second task (singleton search) involved a shape singleton on each trial and measured mechanisms of attention capture and reactive distractor disengagement in (***D***) distractor-absent and (***E***) and distractor-present trials. Although both tasks incorporate a salient color distractor, the search mode required for each task leads to different distractor processing mechanisms.

Participants completed three lab visits and were randomly assigned to one of two groups using custom code (randomization with two groups and stratified by sex at birth). During each lab visit, participants completed the Mindful Attention Awareness Scale (MAAS; Brown and Ryan, 2003) and the Five Facet Mindfulness Questionnaire (FFMQ; Baer et al., 2006). Group A completed the mindfulness meditation intervention between lab visits 1 and 2, and Group B completed the control intervention between lab visits 2 and 3 (Fig. 1*A*). During the active control intervention period, participants listened to an audio recording of a book (as in Zeidan et al., 2010, 2015). This experimental design allowed us to investigate the effects of mindfulness meditation training within-subjects while also counterbalancing the order of the intervention. During each lab visit, participants completed two oculomotor visual search tasks that evoke proactive mechanisms of distractor suppression (Fig. 1*B*,*C*) and reactive disengagement of salient distractors (Fig. 1*D*,*E*). Measuring eye movements during visual search allows assessment of the initiation, scanning, and verification time for attention processing and also interactions between top-down versus bottom-up competing signals that cannot be distinguished from behavioral response times (Hollingworth and Bahle, 2020). In a prior study utilizing these two paradigms, adults made fewer goal-oriented saccades to the target, made more reflexive saccades to the salient distractor when reactively disengaging but not when proactively inhibiting, and had longer dwell times and mean saccadic reaction times compared with young adults (Kim et al., 2024b). These measures of selective attention assess top-down attentional control, distractibility, orienting and reorienting attention, and the timing in which eye movements are initiated which have been shown to be modulated by noradrenaline activity (Morrison and Foote, 1986; Clark et al., 1989; Coull et al., 2001; Dalley et al., 2001; Sara and Bouret, 2012; Snyder et al., 2012; Chamberlain and Robbins, 2013; Logemann et al., 2014; Bouret and Richmond, 2015; Lee et al., 2018, 2020; Bari et al., 2020; Ghosh and Maunsell, 2024). During each lab visit, participants completed the practice for Task 1 (visual search requiring proactive distractor inhibition), three runs of Task 1, completed the practice for Task 2 (visual search requiring reactive distractor disengagement), and three runs of Task 2 (the order for Tasks 1 and 2 was counterbalanced across participants).

At the end of each lab visit, participants were instructed to either complete the guided mindfulness training on the Headspace mobile application or to listen to chapters of the novel *The Adventures of Pinocchio* by Carlo Collodi (free audiobook recording in the public domain from LibriVox; we originally proposed to use the novel *The Adventures of Huckleberry Finn* by Mark Twain but realized some language in the novel could be distressing to participants, undermining the study. Thus, we reached out to the editor and received confirmation to switch audiobooks after Stage 1 in-principle acceptance). Headspace is the highest rated and most popular application for practicing meditation on a mobile device (Mani et al., 2015; Mrazek et al., 2019; O’Daffer et al., 2022) and has demonstrated positive benefits for mental health in numerous mindfulness meditation intervention studies (Bennike et al., 2017; Champion et al., 2018; Economides et al., 2018; Kubo et al., 2018; Noone and Hogan, 2018; Björkstrand et al., 2019; Bostock et al., 2019; Flett et al., 2019). The benefits of mindfulness meditation training using the Headspace application have been validated after brief time periods (less than 1 week of use; Tang et al., 2007; Zeidan et al., 2010; Howarth et al., 2019) and is easy to use and accessible for older adults (Mani et al., 2015; Champion et al., 2018; Mahlo and Windsor, 2021; Burgess et al., 2022). The 30 d intervention time period was chosen given its greater magnitude of benefits compared with 10 d of intervention (Champion et al., 2018) but higher participant retention compared with 6 or 8 weeks of practice requirements (Noone and Hogan, 2018; Bostock et al., 2019; O’Daffer et al., 2022). When beginning the guided mindfulness meditation intervention, participants were provided a username and password with 1 month subscription access to the Headspace application and were instructed to complete the “Basics” 30 d course which contains 10–15 min of guided mindfulness meditation daily. This course comprises guided meditation sessions designed by former Buddhist monk Andy Puddicombe and has previously been utilized as an optimal mindfulness intervention in research investigations (Champion et al., 2018; Mahlo and Windsor, 2021). Participants were informed that the experiment team will monitor their progress in the application and that the team would reach out if daily practice was not completed (see Results, Compliance). Previously, participants completing this mindfulness meditation intervention protocol averaged 25 sessions and ∼4 h of application use across 30 d (Mahlo and Windsor, 2021). Participants were dropped from the study if they did not complete at least 15 sessions and complied with experiment protocols. During the active control intervention period, participants were sent one chapter of the book to listen to daily via email. Each audio recording was 7–20 min long to mimic the length of the guided mindfulness meditation recordings. Participants were instructed to not engage in any other task while listening to the book chapter.

To ensure at-home task adherence and quality control, participants were required to complete a brief survey and journal their daily experience immediately following completion of the daily intervention. Participants were provided a link to access an online form. The form asked participants to provide a rating from a scale of 1–10 on the following question: How would you rate the quality of your attention while completing today's intervention? In addition, participants answered short open-ended questions. When completing the mindfulness intervention, participants answered the questions, “What was the mindfulness instructions you received today?” and “How was the experience of listening to the guided meditation?” When completing the active control condition, participants answered the questions, “What happened in today's audiobook chapter in 1–2 sentences?” and “How was the experience of listening to the audiobook chapter?” Once the form was completed, a copy of the form was immediately sent to the investigators. If participants did not complete the survey or their answers showed no adherence to the required daily intervention task, investigators followed up via phone call to the participants the following day.

### Apparatus

All in-lab tasks were completed on a custom-built NZXT desktop computer (NZXT) equipped with MATLAB software (MathWorks) and Psychophysics Toolbox extensions (Brainard, 1997). Stimuli were presented on a Sun Microsystems 4472 (Oracle) CRT monitor (85 Hz refresh rate). The participants viewed the monitor from 70 cm in a dimly lit, soundproof room. Eye tracking was conducted using the EyeLink 1000 Plus system (SR Research) and head position was maintained using a manufacturer-provided chin rest (SR Research).

### Stimuli and task design

Both visual search tasks consisted of a gaze-contingent fixation display, a visual search array, and an intertrial interval (ITI; Fig. 1*B–E*). The fixation display consisted of a fixation cross (0.7° × 0.7° visual angle) at the center of the screen. The fixation display remained on screen until eye position was registered within 1.3° of the center of the fixation cross for a continuous period of 500 ms (Kim and Anderson, 2022; Kim et al., 2024b). The visual search array was presented for 2,000 ms or until a fixation on the target was registered. If a target was not fixated within the timeout limit, the words “Eye Error” appeared in the center of the screen for 500 ms. Lastly, the ITI consisted of a blank screen for 1,000 ms.

The visual search array for Task 1 (feature search) comprised four unique shapes: a circle, diamond, square, and a hexagon. In this task, participants searched for a feature-specific target shape (circle or diamond, counterbalanced across all participants). The length and width of each of the shapes was 4.2° except for the circle with diameter 4.0° for all shapes to have approximately equal area. Each shape was placed at equal intervals along an imaginary circle with a radius of 9.1°. The color of the shapes was red or green. In addition, the color of the distractor on distractor-present trials was counterbalanced across all participants (red distractor among green shapes or green distractor among red shapes). The search array for Task 2 (singleton search) comprised one unique shape among three other identical shapes (one circle among three diamonds or one diamond among three circles). In this task, participants were required to search for the shape singleton or the unique shape among all shapes. The size, location, and color of the stimuli were identical to that of Task 1. Like in Task 1, the target never was the shape with the unique color. Although the design elements of both oculomotor tasks are similar, adopting an attentional template of a specific feature in Task 1 (feature search mode) leads to suppression of the salient distractor while searching for the unique singleton shape (singleton detection mode) in Task 2 leads to attention capture by the salient distractor (Kim et al., 2024b). As in Task 1, goal-directed selection of a specific feature has shown to “override” stimulus-driven capture by a salient distractor (Bacon and Egeth, 1994), suggesting that maintaining an attentional template for a specific feature map allows prevention of capture by a salient stimulus in an irrelevant feature map. However, this theory has been challenged by studies demonstrating that resistance to capture or distractor rejection is a function of selection history and learning, and not due to differing feature signals (Vatterott and Vecera, 2012; Graves and Egeth, 2015). The precise mechanisms of attention capture and distractor suppression are still debated (Vatterott et al., 2018; De Tommaso and Turatto, 2019; Gaspelin and Luck, 2019; Luck et al., 2021; Lien et al., 2022).

Each run of the visual search tasks contained 96 trials for a total of 288 trials. Trials were distractor present 75% of the time and distractor absent 25% of the time to maximize trial bins in the distractor-present condition. Participants completed a total of 216 distractor-present trials which is more than prior instances of this experiment (Kim et al., 2024b). In distractor-present trials of Task 1, the target shape (circle or diamond) and the location of the distractor with respect to the target was fully counterbalanced. In distractor-present trials of Task 2, the target shape was the circle or diamond equally often and the location of the distractor with respect to the target was fully counterbalanced. In both tasks, the distractor color (red or green) was counterbalanced across participants. In distractor-absent trials of both tasks, the location of the target was fully counterbalanced. Trials were randomized across all participants.

Eye position was calibrated prior to each run using 5-point calibration and was manually drift corrected online by the experimenter as necessary during the gaze-contingent fixation display (Kim and Anderson, 2022; Kim et al., 2024b). During the experiment, the *x*- and *y*-positions of the eyes were continuously monitored in real time with respect to the four stimulus positions, such that fixations were coded online (Kim and Anderson, 2022; Kim et al., 2024b). The EyeLink 1000 Plus system exported an EDF file after each run that contains both sample information collected at 1,000 Hz and manually encoded events for the following: presentation of the fixation stimulus, presentation of the visual search array, fixation of each stimulus during the visual search task, and the end of the trial. Fixation of a stimulus was registered if eye position remains within a region extending 1.0° around the stimulus for a continuous period of at least 50 ms for nontargets and distractors and at least 100 ms for the target (Kim and Anderson, 2022; Kim et al., 2024b). Saccades were defined as a minimum eye velocity threshold of 30° per second and a minimum acceleration threshold of 9,500° per second.

### Proposed analyses pipeline

For both visual search tasks, we measured accuracy, fixation times, first-saccade destinations, dwell times, and mean saccadic reaction times. Accuracy was defined as the percentage of trials in which a fixation was made on the target. Fixation times were defined as the time from the onset of the visual search array until a valid fixation was registered to the target shape. Fixation times that exceed three standard deviations of the mean for a given condition for each participant were trimmed (Kim and Anderson, 2022; Kim et al., 2024b), and fixation times <50 ms were excluded as anticipatory saccades (Gaspelin et al., 2017; Kim and Anderson, 2022; Stilwell et al., 2022). First fixations were defined as the initial stimulus that was registered as a fixation on each trial. First fixations to the target, nontarget, and distractor in addition to fixation times were derived from the eye data coded online. Percentage of saccades to the nontarget shapes were corrected to provide a per-item estimate of fixation; on distractor-absent and distractor-present trials, the percentage of saccades will be divided by 3 and 2, respectively. Oculomotor dwell times were defined as the duration that the eyes remained within the fixation window of the stimulus and were also coded online. Mean saccadic reaction times (sRT) were defined as the mean time to initiate a saccade relative to the onset of the visual search array (Kim and Anderson, 2022; Kim et al., 2024b). sRTs were computed offline from the EDF output using the edfmex MATLAB executable file and the “STARTSACC” code string from the event structure.

To determine the effects of the mindfulness intervention on attentional control, we proposed to conduct a 2 × 2 × 2 analysis of variance (ANOVA) over factors age (young vs older adults), intervention (Mindfulness Group A vs Audiobook Group B), and time (Visit 1 vs Visit 2) over each measure (see Hypothesis below). Next, to determine the age-related effects of mindfulness meditation intervention on the aforementioned measures of attentional control, we proposed to conduct a 2 × 2 ANOVA analysis over factors age (young vs older adults) and time (baseline vs post-mindfulness intervention) over each measure (see also below, Exploratory analyses, for identifying practice effects). This analysis used the full sample and comprised Visit 1 to Visit 2 for Group A and Visit 2 to Visit 3 for Group B.

To correct for multiple comparisons in our planned analyses, we used the false discovery rate (FDR) method using adaptive linear step-up procedures (Benjamini et al., 2006; Glickman et al., 2014). We conducted six unique ANOVA analyses for each hypothesis (Hypothesis 1: Mindfulness meditation will improve attentional control; Hypothesis 2: Older adults will show greater benefits from the intervention compared with young adults) and corrected at FDR *α* = 0.05. *p* values were adjusted from the two-state Benjamini and Hochberg linear step-up procedure ('TSBH”) using the R package, “multtest” (version 2.54.0) for these 12 hypothesis-driven analyses.

We did not predict any improvements in accuracy and proactive oculomotor suppression as performance is near ceiling in these visual search tasks in both young and older adults (Kim et al., 2024b). We used accuracy as an indicator of data quality, and participants’ run data that do not attain at least 60% accuracy over all visits were proposed to be omitted as poor data quality during acquisition. Although we had two subjects (older adults) who had accuracy below 60% for one visit, these subjects did not have overall accuracy <60% over all visits or had significantly lower accuracy relative to other subjects, suggesting that this low accuracy is just because of the performance of the participant and not because of poor data quality during acquisition. Therefore, we included the data for these older adult subjects.

Given that the distractor is proactively suppressed in the feature search task, we identified some subjects did not look at the distractor at all or looked at it very few times. This sometimes resulted in nonphysiologically plausible data that were likely caused by eye tracking errors due to 1 or 2 data points. Thus, we did not include data for mean saccadic reaction times or dwell times to the distractor in which the total number of first fixations to the distractor (for that visit accumulated over 3 runs) was two or fewer times and if the time was <100 ms (Visit 1 = 8 subjects, Visit 2 = 7 subjects, Visit 3 = 5 subjects). For reference, saccadic reaction times <50 ms were already removed, given that they were deemed as anticipatory. The data for these subjects were excluded only for the dwell time and saccadic reaction time measures. For full transparency, statistical conclusions did not change even when including these outliers.

#### Hypotheses

Mindfulness meditation will improve mechanisms of attentional control. Older adults will show greater benefits from the mindfulness intervention compared with young adults. We tested our hypotheses on the following six measures of attentional control that are impaired in older adults (Kim et al., 2024b).

- Distractibility: Attention capture by salient, task-irrelevant distractor (1) will be decreased.
- Goal-directed attentional control: Percentage of first saccades toward the target will be increased in both distractor-absent (2) and distractor-present trials (3).
- Attention processing: Fixation times (4) and mean saccadic reaction times (5) will be decreased.
- Reactive disengagement: Dwell time on initial incorrect fixations (6) will be decreased.

### Exploratory analyses

We planned to conduct exploratory analyses that are not central to our hypothesis but of interest, given our experimental design. First, we correlated measures acquired on Visit 1 (baseline) and the visit following the control intervention (either visits 2 or 3 depending on the assigned group) to determine test–retest reliability of oculomotor mechanisms of attention. In addition, we proposed to determine whether the benefits of 30 d of mindfulness meditation practice persist among participants in Group A by comparing data from Lab Visit 3 to that of Lab Visit 2. This analysis would be important in observing potential practice effects and to determine whether the extra visit before the intervention contributes to elevated performance in Group B. Furthermore, we conducted the aforementioned ANOVA analyses over the oculomotor suppression effect to investigate whether mindfulness meditation impairs this aspect of attentional control, contrary to our hypotheses (we predicted a null effect). Last, we conducted the aforementioned ANOVA analyses between middle-aged older adults aged 50–65 years old and older adults aged 65–80 years old to determine whether older adults aged 65–80 years old showed greater locus ceruleus dysfunction compared with middle-aged older adults.

### Transparency and openness

The preregistered manuscript, raw data, and materials have been uploaded to the Open Science Framework, osf.io/u3b8n.

## Results

For each mechanism of attentional control, we first show the results of the feature search task (proactive suppression of the salient distractor; Fig. 1*B*,*C*) followed by the results from the singleton search task (reactive disengagement of the salient distractor; Fig. 1*D*,*E*).

### Preregistered analyses 1: combined ANOVA over Visits 1 and 2

We conducted a 2 × 2 × 2 mixed ANOVA over factors age (Young vs Older), intervention (Mindfulness vs Audiobook), and time (Visit 1 vs Visit 2) over each dependent measure. Although this analysis does not make use of the third time point, we conducted this analysis as preregistered. If we identified a significant main effect of age, we also conducted the aforementioned ANOVA with three levels for the age factor (Young vs Middle-Aged vs Older) and report the main effect of age and any significant interactions. We followed up significant main effects of age in this ANOVA comprising three levels for the age factor with post hoc *t* tests using Tukey HSD multiple-comparison corrections. In this first analysis, a significant time × intervention interaction would show that the mindfulness meditation intervention improved mechanisms of attentional control that is not observable following the positive control, audiobook intervention.

#### Distractibility—first saccade to salient distractor

For the feature search task, we observed significant main effects of age, *F*_(1,64)_ = 13.73, *p* < 0.001, *η_p_*^2^ = 0.177, and time, *F*_(1,64)_ = 5.98, *p* = 0.017, *η_p_*^2^ = 0.086, but no significant main effect of intervention, *F*_(1,64)_ = 0.17, *p* = 0.686, nor a significant time × intervention interaction, *F*_(1,64)_ = 0.31, *p* = 0.580. In the 3 × 2 × 2 ANOVA, we observed a significant main effect of age, *F*_(2,62)_ = 6.65, *p* = 0.002, *η_p_*^2^ = 0.177. Young adults exhibited less distractibility compared with middle-aged adults, *p* = 0.012 (mean diff = 4.80, SE = 1.63, 95% CI [−8.70 −0.90]), and older adults, *p* = 0.005 (mean diff = 5.28, SE = 1.63, 95% CI [−9.19 −1.38]), but no significant differences were observed between the middle-aged and older adults, *p* = 0.959 (mean diff = 0.48, SE = 1.76, 95% CI [−4.70 3.73]). To appropriately measure attention suppression in this visual search task (Wöstmann et al., 2022), we calculated the oculomotor suppression effect as a measure of increased saccades to the salient distractor compared with a within-subjects control of reflexive saccades to nontarget stimuli (oculomotor suppression = first saccades to nontarget stimuli − first saccades to distractor). Thus, a positive value indicates increased proactive suppression of the distractor. In the 2 × 2 × 2 ANOVA, we observed no significant main effects of age, *F*_(1,64)_ = 0.06, *p* = 0.809, time, *F*_(1,64)_ = 0.69, *p* = 0.409, nor intervention, *F*_(1,64)_ = 0.81, *p* = 0.371, nor time × intervention interaction, *F*_(1,64)_ = 0.08, *p* = 0.784.

For the singleton search task, in the 2 × 2 × 2 ANOVA, we observed a significant main effect of age, *F*_(1,65)_ = 11.04, *p* = 0.001, *η_p_*^2^ = 0.145, and time, *F*_(1,65)_ = 9.58, *p* = 0.003, *η_p_*^2^ = 0.128, but no significant main effect of intervention, *F*_(1,65)_ = 0.04, *p* = 0.846, nor a significant time × intervention interaction, *F*_(1,65)_ = 0.15, *p* = 0.697. In the 3 × 2 × 2 ANOVA, we observed a significant main effect of age, *F*_(2,63)_ = 6.90, *p* = 0.002, *η_p_*^2^ = 0.180. Young adults exhibited less distractibility compared with older adults, *p* = 0.001 (mean diff = 12.53, SE = 3.34, 95% CI [−20.55 −4.51]), but no differences between young and middle-aged adults, *p* = 0.143 (mean diff = −6.48, SE = 3.39, 95% CI [−14.62 1.65], and middle-aged and older adults, *p* = 0.224 (mean diff = 6.04, SE = 3.62, 95% CI [−2.64 14.72]). In this task we calculated the oculomotor capture effect as a measure of increased saccades to the salient distractor compared with a within-subjects control of reflexive saccades to nontarget stimuli (oculomotor capture = first saccades to distractor − first saccades to nontarget stimuli). Thus, a positive value indicates increased distractibility by the distractor. In the 2 × 2 × 2 ANOVA, we observed no significant main effects of age, *F*_(1,65)_ = 1.99, *p* = 0.164, time, *F*_(1,65)_ = 3.81, *p* = 0.055, intervention, *F*_(1,65)_ = 0.06, *p* = 0.815, nor a time × intervention interaction, *F*_(1,65)_ = 0.22, *p* = 0.640.

#### Goal-directed attentional control in distractor-absent trials—first saccade to target

For the feature search task, in the 2 × 2 × 2 ANOVA, we observed significant main effects of age, *F*_(1,64)_ = 17.69, *p* < 0.001, *η_p_*^2^ = 0.217, and time, *F*_(1,64)_ = 5.45, *p* = 0.023, *η_p_*^2^ = 0.078, but no significant main effect of intervention, *F*_(1,64)_ = 2.25, *p* = 0.139. We did not observe a significant time × intervention interaction, *F*_(1,64)_ = 1.69, *p* = 0.198. In the 3 × 2 × 2 ANOVA, we observed a significant main effect of age, *F*_(2,62)_ = 8.72, *p* < 0.001, *η_p_*^2^ = 0.220. Young adults exhibited greater goal-directed attentional control compared with middle-aged adults, *p* = 0.006 (mean diff = 12.33, SE = 3.85, 95% CI [3.10 21.58]), and older adults, *p* < 0.001 (mean diff = 14.81, SE = 3.85, 95% CI [5.57 24.05]), but no differences between the middle-aged and older adults, *p* = 0.823 (mean diff = 2.47, SE = 4.16, 95% CI [−7.51 12.45]).

For the singleton search task, in the 2 × 2 × 2 ANOVA, we observed a significant main effect of age, *F*_(1,65)_ = 33.82, *p* < 0.001, *η_p_*^2^ = 0.342, but no significant main effects of intervention, *F*_(1,65)_ = 3.13, *p* = 0.082, nor time, *F*_(1,65)_ = 1.59, *p* = 0.211. We did not observe a significant time × intervention interaction, *F*_(1,65)_ = 1.30, *p* = 0.258. In the 3 × 2 × 2 ANOVA, we observed a significant main effect of age, *F*_(2,63)_ = 28.54, *p* < 0.001, *η_p_*^2^ = 0.475. Young adults exhibited greater goal-directed attentional control compared with middle-aged adults, *p* = 0.004 (mean diff = 9.21, SE = 2.84, 95% CI [2.40 16.02]), and older adults, *p* < 0.001 (mean diff = 21.26, SE = 2.80, 95% CI [14.54 27.98]). Furthermore, middle-aged adults exhibited greater goal-directed attentional control compared with older adults, *p* < 0.001 (mean diff = 12.05, SE = 3.03, 95% CI [4.78 19.32]).

#### Goal-directed attentional control in distractor-present trials—first saccade to target

For the feature search task, in the 2 × 2 × 2 ANOVA, we observed significant main effects of age, *F*_(1,64)_ = 30.02, *p* < 0.001, *η_p_*^2^ = 0.319, and time, *F*_(1,64)_ = 12.07, *p* < 0.001, *η_p_*^2^ = 0.159, but no significant main effect of intervention, *F*_(1,64)_ = 2.40, *p* = 0.126. We did not find a significant time × intervention interaction, *F*_(1,64)_ = 0.48, *p* = 0.491. In the 3 × 2 × 2 ANOVA, we observed a significant main effect of age, *F*_(2,62)_ = 15.19, *p* < 0.001, *η_p_*^2^ = 0.329. Young adults exhibited greater goal-directed attentional control compared with middle-aged adults, *p* < 0.001 (mean diff = 14.00, SE = 3.44, 95% CI [5.73 22.27]), older adults, *p* < 0.001 (mean diff = 17.78, SE = 3.44, 95% CI [9.50 26.05]), but no differences between middle-aged and older adults, *p* = 0.571 (mean diff = 3.77, SE = 3.72, 95% CI [−5.16 12.71]).

For the singleton search task, in the 2 × 2 × 2 ANOVA, we observed a significant main effect of age, *F*_(1,65)_ = 38.44, *p* < 0.001, *η_p_*^2^ = 0.372, and time, *F*_(1,65)_ = 15.75, *p* < 0.001, *η_p_*^2^ = 0.195, but no significant main effects of intervention, *F*_(1,65)_ = 0.94, *p* = 0.335. We did not find a significant time × intervention interaction, *F*_(1,65)_ = 0.00, *p* = 0.982, but identified a significant time × age interaction, *F*_(1,65)_ = 4.66, *p* = 0.035, *η_p_*^2^ = 0.067. In the 3 × 2 × 2 ANOVA, we observed a significant main effect of age, *F*_(2,63)_ = 24.78, *p* < 0.001, *η_p_*^2^ = 0.440. Young adults exhibited greater goal-directed attentional control compared with middle-aged adults, *p* < 0.001 (mean diff = 14.32, SE = 3.68, 95% CI [5.50 23.14]), older adults, *p* < 0.001 (mean diff = 25.43, SE = 3.62, 95% CI [16.73 34.12]). Furthermore, middle-aged adults exhibited greater goal-directed attentional control compared with older adults, *p* = 0.017 (mean diff = 11.11, SE = 3.92, 95% CI [1.69 20.52]).

#### Attention processing—fixation times in distractor-present trials

For the feature search task, in the 2 × 2 × 2 ANOVA, we observed a significant main effect of age, *F*_(1,64)_ = 21.40, *p* < 0.001, *η_p_*^2^ = 0.251, but no significant main effects of time, *F*_(1,64)_ = 2.76, *p* = 0.100, nor intervention, *F*_(1,64)_ = 0.67, *p* = 0.418. We did not find a significant time × intervention interaction, *F*_(1,64)_ = 1.75, *p* = 0.19. In the 3 × 2 × 2 ANOVA, we observed a significant main effect of age, *F*_(2,62)_ = 12.32 *p* < 0.001, *η_p_*^2^ = 0.284. Young adults were faster to find the target compared with middle-aged adults, *p* = 0.011 (mean diff = −61.88, SE = 20.62, 95% CI [−111.37 −12.38]), and older adults, *p* < 0.001 (mean diff = −98.87, SE = 20.62, 95% CI [−148.37 −49.38]). However, we did not observe significant differences between middle-aged and older adults, *p* = 0.228 (mean diff = −37.00, SE = 22.26, 95% CI [−90.46 16.46]).

For the singleton search task, in the 2 × 2 × 2 ANOVA, we observed a significant main effect of age, *F*_(1,65)_ = 41.43, *p* < 0.001, *η_p_*^2^ = 0.389, and time, *F*_(1,65)_ = 7.16, *p* = 0.009, *η_p_*^2^ = 0.099, but no significant main effect of intervention, *F*_(1,65)_ = 2.10, *p* = 0.152. We did not find a significant time × intervention interaction, *F*_(1,65)_ = 0.19, *p* = 0.669. In the 3 × 2 × 2 ANOVA, we observed a significant main effect of age, *F*_(2,63)_ = 22.43, *p* < 0.001, *η_p_*^2^ = 0.416. Young adults were faster compared with middle-aged adults, *p* < 0.001 (mean diff = −149.10, SE = 32.30, 95% CI [−226.64 −71.56]), and older adults, *p* < 0.001 (mean diff = −200.72, SE = 31.85, 95% CI [−277.18 −124.26]). However, there were no significant differences between middle-aged and older adults, *p* = 0.299 (mean diff = −51.62, SE = 34.48, 95% CI [−134.37 31.13]).

#### Attention processing—saccadic reaction times to distractor

For the feature search task, in the 2 × 2 × 2 ANOVA, we observed no significant main effects of age, *F*_(1,51)_ = 0.99, *p* = 0.325, time, *F*_(1,51)_ = 0.37, *p* = 0.548, intervention, *F*_(1,51)_ = 0.11, *p* = 0.738, nor a time × intervention interaction, *F*_(1,51)_ = 3.37, *p* = 0.072.

For the singleton search task in the 2 × 2 × 2 ANOVA, we did not observe significant main effects of age, *F*_(1,65)_ = 1.67, *p* = 0.201, nor time, *F*_(1,65)_ = 1.19, *p* = 0.279, but found a significant main effect of intervention, *F*_(1,65)_ = 4.24, *p* = 0.044, *η_p_*^2^ = 0.061. We did not find a significant time × intervention interaction, *F*_(1,65)_ = 2.74, *p* = 0.103, but observed a significant time × age interaction, *F*_(1,65)_ = 8.19, *p* = 0.006, *η_p_*^2^ = 0.112. In post hoc *t* test analyses, young adults showed faster saccadic reaction times in Visit 2 compared with Visit 1, *t*_(27)_ = 2.70, *p* = 0.012, *d* = 0.515 (Visit 1: Mean = 288.01, SE = 6.97; Visit 2: Mean = 269.85, SE = 6.32). However, we found no evidence for changes in older adults, *t*_(40)_ = 1.31, *p* = 0.196 (Visit 1: Mean = 286.66, SE = 7.58; Visit 2: Mean = 294.70.85, SE = 6.32). In the 3 × 2 × 2 ANOVA, we did not observe a significant main effect of age, *F*_(2,63)_ = 0.89, *p* = 0.417, but again identified a significant main effect of intervention, *F*_(1,63)_ = 7.84, *p* = 0.007, *η_p_*^2^ = 0.111, and a significant time × age interaction, *F*_(2,63)_ = 4.24, *p* = 0.019, *η_p_*^2^ = 0.119.

#### Reactive disengagement—dwell times on distractor

For the feature search task, in the 2 × 2 × 2 ANOVA, we observed no significant main effects of age, *F*_(1,57)_ = 0.05, *p* = 0.829, time, *F*_(1,57)_ = 0.49, *p* = 0.489, intervention, *F*_(1,57)_ = 0.85, *p* = 0.360, nor a time × intervention interaction, *F*_(1,57)_ = 0.86, *p* = 0.358.

For the singleton search task, in the 2 × 2 × 2 ANOVA, we observed a significant main effect of age, *F*_(1,65)_ = 28.89, *p* < 0.001, *η_p_*^2^ = 0.308, but no main effects of time, *F*_(1,65)_ = 0.00, *p* = 0.961, nor intervention, *F*_(1,65)_ = 2.17, *p* = 0.145. In addition, we observed a significant three-way age × time × intervention interaction, *F*_(1,65)_ = 6.06, *p* = 0.017, *η_p_*^2^ = 0.085. However, this three-way interaction was not significant when correcting for multiple comparisons, *p*_adj_ = 0.102. In the 3 × 2 × 2 ANOVA, we again observed a significant main effect of age, *F*_(2,63)_ = 13.99, *p* < 0.001, *η_p_*^2^ = 0.308. Young adults exhibited shorter dwell times compared with middle-aged adults, *p* < 0.001 (mean diff = −59.76, SE = 13.41, 95% CI [−91.94 −27.58]), and older adults, *p* < 0.001 (mean diff = −58.69, SE = 13.22, 95% CI [−90.42 −26.96]).

In summary, from this first analysis without using the Visit 3 part of our data, we did not identify evidence to support our hypothesis that mindfulness meditation improves mechanisms of attentional control after correcting for multiple comparisons. However, we identified multiple main effects of age when using three levels (young vs middle-aged vs older). Thus, we used three levels for the age factor in Analysis 2.

### Preregistered analysis 2: mixed ANOVA analyses over the mindfulness meditation intervention period with the full sample

We conducted a 3 × 2 mixed ANOVA with factors age (young vs middle-aged vs older) and time (baseline vs post-intervention) over the mindfulness meditation intervention combined for both groups (Group A: Visit 1 to Visit 2, Group B: Visit 2 to Visit 3). To investigate Hypothesis 1, we assessed whether the main effect of time was significant to determine whether mindfulness meditation improved mechanisms of attentional control. To investigate Hypothesis 2, we assessed whether the age × time interaction was significant to determine whether the effects of mindfulness meditation differed among age groups. Although we still report the oculomotor capture and oculomotor suppression effects as within-subjects controlled measures of attention capture and inhibition of the salient distractor, we used the six preregistered measures and *p* values in our multiple-comparison corrections and final conclusions.

#### Distractibility—first saccade to salient distractor

**Figure 2.**
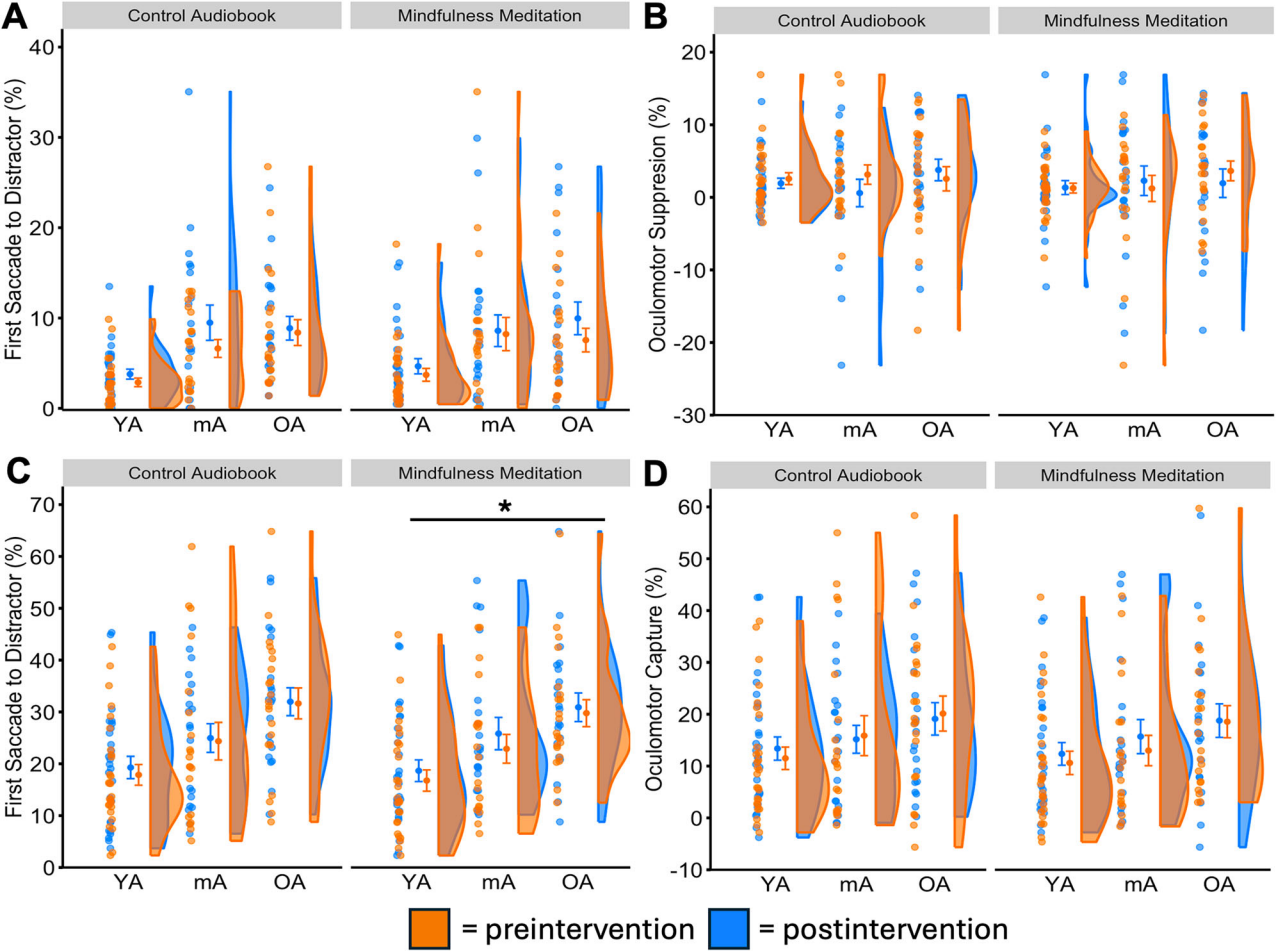
Effects on attention capture by the salient distractor. In the feature search task, the salient distractor is proactively suppressed. We did not find evidence that mindfulness meditation modulates (***A***) first saccades to the distractor and the (***B***) oculomotor suppression effect. In the singleton search task, the salient distractor is designed to be reactively disengaged after fixation. Here, we found evidence that mindfulness meditation improves task-irrelevant distractibility with (***C***) first saccades to the distractor and the (***D***) oculomotor capture effect. Asterisks show statistical significance in the main effect of the mindfulness meditation intervention in the 3 × 2 mixed ANOVA. For first saccades to the distractor, we show the corrected *p* value following multiple-comparison corrections. *p_adj_ < 0.05, YA, young adults; mA, middle-aged adults; OA, older adults. See Extended Data Figure 2-1 for descriptive statistics.

In the feature search task, over the first saccade to distractor measure, we identified a significant main effect of age, *F*_(2,64)_ = 5.52, *p* = 0.006, *η_p_*^2^ = 0.105, but no main effect of time, *F*_(1,64)_ = 2.07, *p* = 0.155, nor interaction, *F*_(2,64)_ = 0.45, *p* = 0.637 (Fig. 2*A*). With the oculomotor suppression effect as the dependent measure, we again did not identify a main effect of age, *F*_(2,64)_ = 0.29, *p* = 0.753, time, *F*_(1,64)_ = 0.01, *p* = 0.923, nor interaction, *F*_(2,64)_ = 0.57, *p* = 0.569 (Fig. 2*B*).

10.1523/ENEURO.0356-23.2025.f2-1 Figure 2-1 Extended data table supporting Figure 2 with descriptive statistics. All values are presented as means and standard errors in parentheses. Download Figure 2-1, DOCX file.

In the singleton search task, over the first saccade to distractor measure, we identified significant main effects of age, *F*_(2,65)_ = 7.03, *p* = 0.002, *η_p_*^2^ = 0.178, and time, *F*_(1,65)_ = 7.66, *p* = 0.007, *η_p_*^2^ = 0.105, but no interaction effect, *F*_(2,65)_ = 0.49, *p* = 0.616 (Fig. 2*C*). Over the oculomotor capture effect, we identified a marginal main effect of time, *F*_(1,65)_ = 4.00, *p* = 0.050, *η_p_*^2^ = 0.058, and no main effect of age, *F*_(2,65)_ = 1.93, *p* = 0.168, nor an interaction effect, *F*_(2,65)_ = 0.81, *p* = 0.45 (Fig. 2*D*).

#### Goal-directed attentional control in distractor-absent trials—first saccade to target

**Figure 3.**
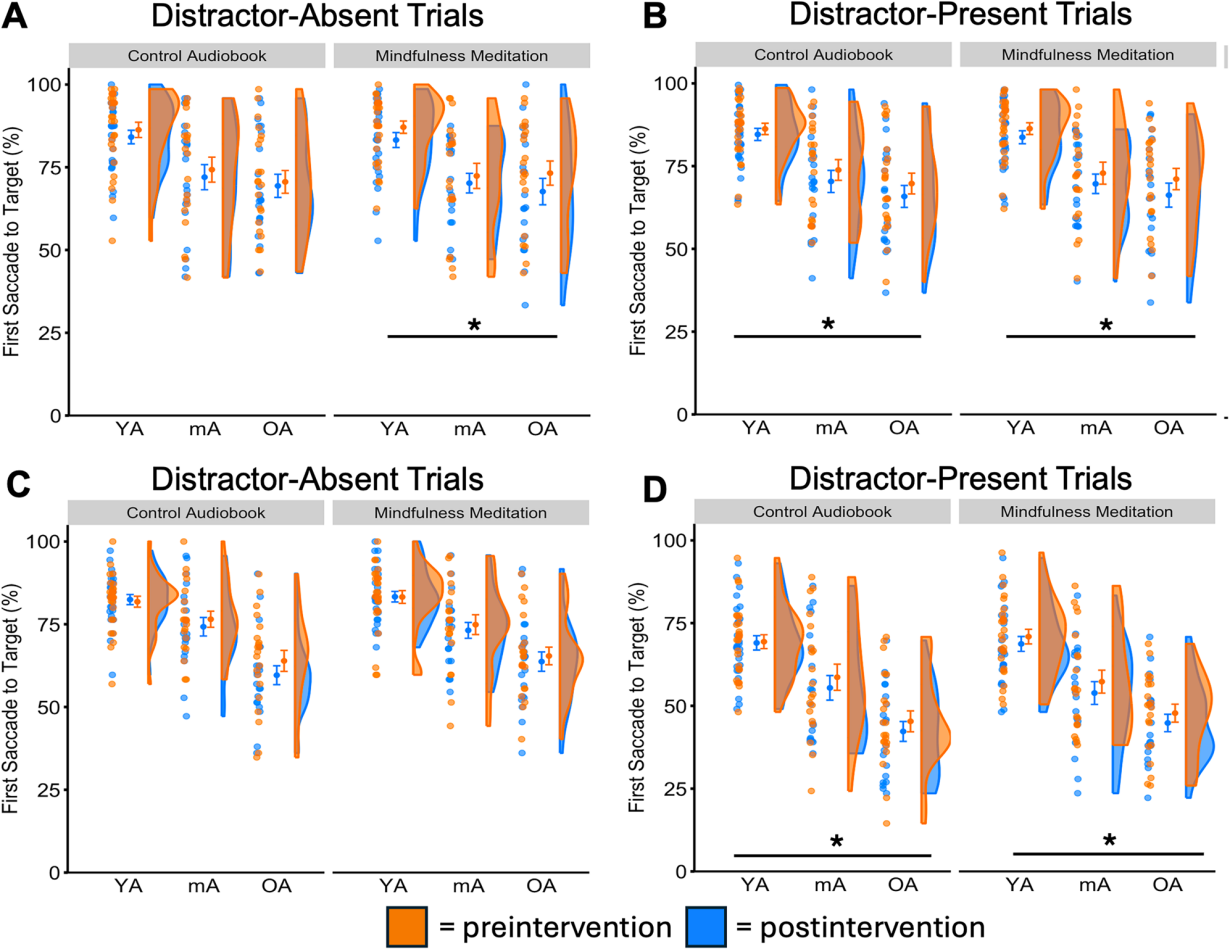
Effects on goal-directed attentional control. In the feature search task, we identified that mindfulness meditation increases first saccades to the target shape in (***A***) distractor-absent and (***B***) distractor-present trials. Interestingly, goal-directed attentional control in distractor-present trials also improved in the audiobook (positive control) condition. In the singleton search task, goal-directed attentional control did not improve in either condition in (***C***) distractor-absent trials but improved for both conditions in (***D***) distractor-present trials. Asterisks show statistical significance in the main effect of each intervention in the 3 × 2 mixed ANOVA and we indicate the corrected *p* values following multiple-comparison corrections. *p_adj_ < 0.05, YA, young adults; mA, middle-aged adults; OA, older adults. See Extended Data Figure 3-1 for descriptive statistics.

In the feature search task, we identified a significant main effect of time, *F*_(1,64)_ = 8.34, *p* = 0.005, *η_p_*^2^ = 0.115, and age, *F*_(2,64)_ = 9.53, *p* < 0.001, *η_p_*^2^ = 0.230, but no interaction, *F*_(2,64)_ = 0.49, *p* = 0.618 (Fig. 3*A*). In the singleton search task, we identified a main effect of age, *F*_(2,65)_ = 19.22, *p* < 0.001, *η_p_*^2^ = 0.372, but no main effect of time, *F*_(1,65)_ = 0.93, *p* = 0.338, nor interaction, *F*_(2,65)_ = 0.30, *p* = 0.745 (Fig. 3*C*).

10.1523/ENEURO.0356-23.2025.f3-1 Figure 3-1 Extended data table supporting Figure 3 with descriptive statistics. All values are presented as means and standard errors in parentheses. Download Figure 3-1, DOCX file.

#### Goal-directed attentional control in distractor-present trials—first saccade to target

In the feature search task, like in distractor-absent trials, we again identified a significant main effect of age, *F*_(2,64)_ = 13.31, *p* < 0.001, *η_p_*^2^ = 0.294, and time, *F*_(1,64)_ = 7.83, *p* = 0.007, *η_p_*^2^ = 0.109, but no interaction, *F*_(2,64)_ = 0.28, *p* = 0.754 (Fig. 3*B*). In the singleton search task, during the mindfulness meditation period, we identified a significant main effect of age, *F*_(2,65)_ = 22.34, *p* < 0.001, *η_p_*^2^ = 0.294, time, *F*_(1,65)_ = 7.52, *p* = 0.008, *η_p_*^2^ = 0.104, but no interaction, *F*_(2,65)_ = 0.13, *p* = 0.883 (Fig. 3*D*).

#### Attention processing—fixation times in distractor-present trials

**Figure 4.**
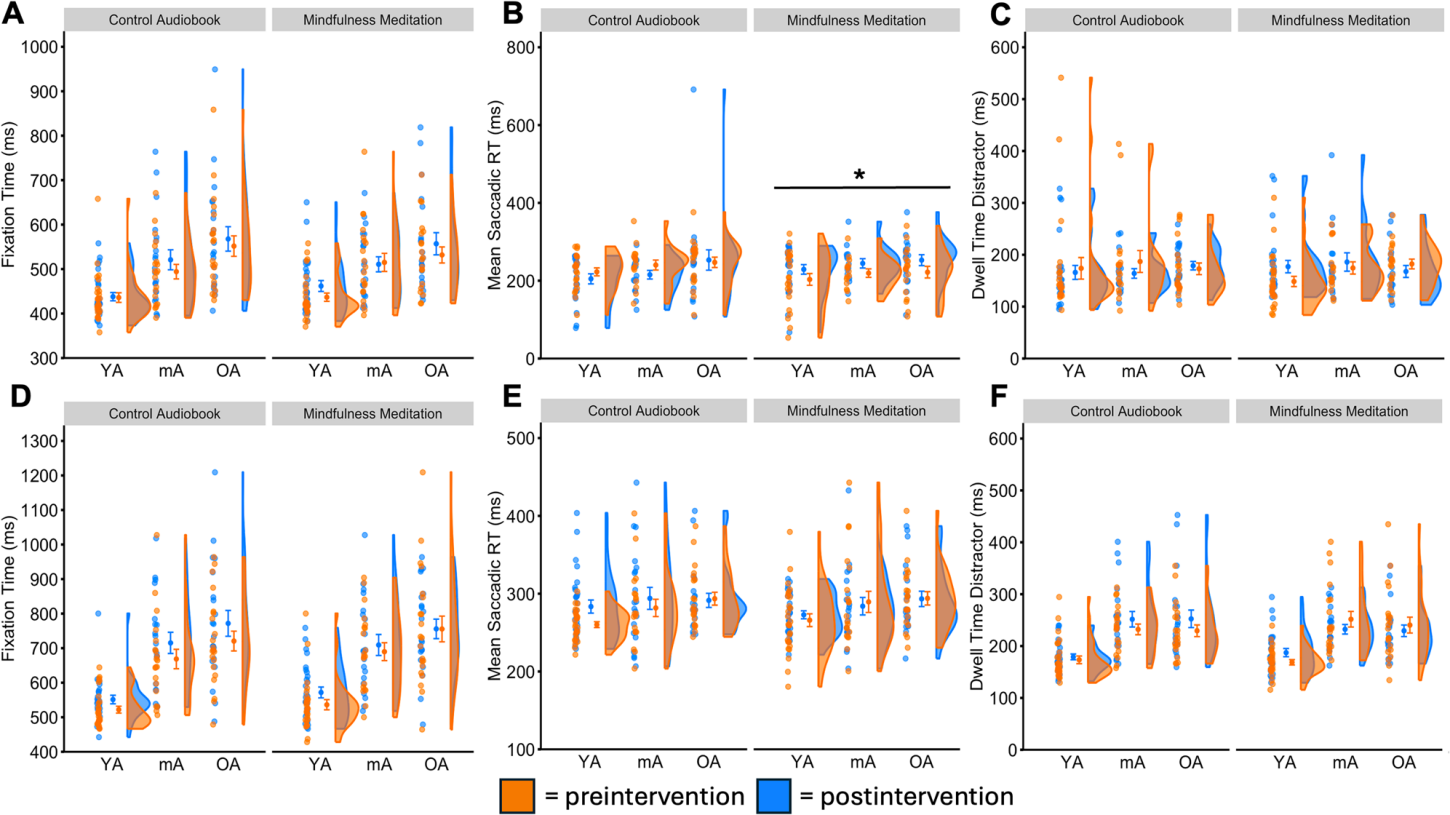
Effects on attention processing speeds. We measured three mechanisms of attentional control that assess processing speeds: fixation times as a proxy of orienting and reorienting, mean saccadic reaction times as a proxy of saccade initiation, and dwell times as a proxy of disengagement after stimulus rejection. In the feature search task, we identified that mindfulness meditation (***A***) did not modulate overall fixation times, (***B***) improved saccadic reaction times, and (***C***) did not modulate dwell times. In the singleton search task, we identified that mindfulness meditation did not modulate (***D***) fixation times, (***E***) saccadic reaction times, or (***F***) dwell times. Asterisks indicate statistical significance in the main effect of each intervention in the 3 × 2 mixed ANOVA and we indicate the corrected *p* values following multiple-comparison corrections. *p_adj_ < 0.05, YA, young adults; mA, middle-aged adults; OA, older adults. See Extended Data Figure 4-1 for descriptive statistics.

In the feature search task, we identified a significant main effect of age, *F*_(2,64)_ = 11.83, *p* < 0.001, *η_p_*^2^ = 0.270, but no main effect of time, *F*_(1,64)_ = 2.63, *p* = 0.110, nor interaction, *F*_(2,64)_ = 1.03, *p* = 0.362 (Fig. 4*A*). In the singleton search task, we identified a significant main effect of age, *F*_(2,65)_ = 21.91, *p* < 0.001, *η_p_*^2^ = 0.403, but no main effect of time, *F*_(1,65)_ = 2.60, *p* = 0.112, nor interaction, *F*_(2,65)_ = 0.85, *p* = 0.433 (Fig. 4*D*).

10.1523/ENEURO.0356-23.2025.f4-1 Figure 4-1 Extended data table supporting Figure 4 with descriptive statistics. All values are presented as means and standard errors in parentheses. Download Figure 4-1, DOCX file.

#### Attention processing—saccadic reaction times to distractor

In the feature search task, we identified a significant main effect of time, *F*_(1,52)_ = 5.98, *p* = 0.018, *η_p_*^2^ = 0.103, but no main effects of age, *F*_(2,52)_ = 1.31, *p* = 0.279, nor interaction, *F*_(2,52)_ = 0.03, *p* = 0.973 (Fig. 4*B*). In the singleton search task, we did not identify significant main effects of age, *F*_(2,65)_ = 2.35, *p* = 0.104, time, *F*_(1,65)_ = 0.00, *p* = 0.96, nor interaction, *F*_(2,65)_ = 0.67, *p* = 0.517 (Fig. 4*E*).

#### Reactive disengagement—dwell times on distractor

In the feature search task, we did not identify significant main effects of age, *F*_(2,58)_ = 0.92, *p* = 0.404, time, *F*_(1,58)_ = 1.00, *p* = 0.322, nor interaction, *F*_(2,58)_ = 2.39, *p* = 0.101 (Fig. 4*C*). In the singleton search task, we identified a significant main effect of age, *F*_(2,65)_ = 14.90, *p* < 0.001, *η_p_*^2^ = 0.314, did not observe a main effect of time, *F*_(1,65)_ = 0.47, *p* = 0.495, but found a significant interaction effect, *F*_(2,65)_ = 4.43, *p* = 0.016, *η_p_*^2^ = 0.120 (Fig. 4*F*).

As preregistered, we applied the FDR method with Benjamini–Hochberg linear step-up method to correct for multiple comparisons. For Hypothesis 1 (effect of mindfulness meditation), we used the preregistered measures’ *p* values over the main effect of time. For Hypothesis 2 (age differences), we used the *p* values over the age × time interaction effect. In summary, in the feature search task, we identified that mindfulness meditation improves goal-directed attentional control in distractor-absent trials (*p* = 0.005, *p*_adj_ = 0.011), distractor-present trials (*p* = 0.007, *p*_adj_ = 0.011), and saccadic reaction times (*p* = 0.018, *p*_adj_ = 0.018). No age-related interactions were significant. In the singleton search task, we identified that mindfulness meditation improves distractibility (*p* = 0.007, *p*_adj_ = 0.016) and goal-directed attentional control in distractor-present trials (*p* = 0.008, *p*_adj_ = 0.016). Although we identified a significant time × age interaction with dwell time as the dependent measure, the interaction was not significant after multiple-comparison corrections (*p* = 0.016, *p*_adj_ = 0.096).

We also conducted the aforementioned 3 × 2 mixed ANOVA over the audiobook intervention (positive control) period for these measures. In the feature search task, we did not observe a significant main effect of time (nor an interaction effect) over goal-directed attentional control in distractor-absent trials, *F*_(1,65)_ = 2.51, *p* = 0.118, and saccadic reaction times, *F*_(1,54)_ = 1.06, *p* = 0.308, However, we observed a significant main effect over goal-directed attentional control in distractor-present trials, *F*_(1,65)_ = 6.14, *p* = 0.016, *η_p_*^2^ = 0.086. In the singleton search task, we did not observe a significant main effect of time (nor an interaction effect) over distractibility, *F*_(1,65)_ = 0.67, *p* = 0.416, during the audiobook intervention period. Interestingly, we again found a significant main effect of time in goal-directed attentional control in distractor-present trials, *F*_(1,65)_ = 4.59, *p* = 0.036, *η_p_*^2^ = 0.066. Given that both the audiobook and mindfulness meditation intervention improved goal-directed attentional control in distractor-present trials, we investigated whether this increase in performance was because of practice effects. As preregistered, we compared group means among Group A participants in their Visits 2 and 3 to determine whether performance continuously improved the more practice participants had with the task. However, we did not find evidence that goal-directed attentional control in distractor-present trials improved in Visit 3 from Visit 2 when completing the feature search task, *t*_(32)_ = 0.97, *p* = 0.340, and the singleton search task, *t*_(33)_ = 0.07, *p* = 0.942.

Finally, we explored whether practice effects were contributing to the longitudinal intervention benefits and conducted post hoc exploratory ANOVA analyses with factors intervention (mindfulness vs audiobook) and age (young vs middle-aged vs older) over the difference scores between the mindfulness and audiobook interventions (post-pre intervention). In the feature search task, we again identified a significant main effect of intervention, *F*_(1,48)_ = 9.38, *p* = 0.004, *η_p_*^2^ = 0.086, but no main effect of age, *F*_(2,48)_ = 1.51, *p* = 0.232, nor interaction, *F*_(2,48)_ = 0.02, *p* = 0.979, over saccadic reaction times, as in our prior analyses (Fig. 4*B*). However, we did not observe main effects of intervention nor interactions over any other attention mechanisms, including goal-directed attentional control and distractibility by salient stimuli.

### Test–retest reliability

Prior studies have shown high reliability (internal consistency) for the acquired oculomotor measures in this study for both young and older adults (Kim et al., 2024a). Given the longitudinal nature of this study, we investigated the test–retest reliability for these measures before and after the positive control (audiobook) intervention month for Group B, who completed the audiobook first. Our recruited sample showed robust test–retest reliability over measures of attention capture/suppression by a salient distractor and attention processing speeds in both oculomotor search tasks (Table 1).

**Table 1.** Test–retest reliability of oculomotor control measures in both feature search and singleton search tasks

Measure	Pearson's *r*	*p* value
Oculomotor suppression (feature search)	0.796	<0.001
Fixation time (feature search)	0.603	<0.001
Saccadic reaction time (feature search)	0.360	0.037
Oculomotor capture (singleton search)	0.819	<0.001
Fixation time (singleton search)	0.896	<0.001
Saccadic reaction time (singleton search)	0.519	0.001

### Mindfulness questionnaires: MAAS and FFMQ-15

We conducted a 3 × 2 ANOVA over factors age (young vs middle-aged vs older) and time (pre- vs post-intervention) over the guided mindfulness meditation and the audiobook intervention periods. For the MAAS score, over the mindfulness meditation period, we identified a significant main effect of age, *F*_(2,65)_ = 7.37, *p* = 0.001, *η_p_*^2^ = 0.185, but no main effects of time, *F*_(1,65)_ = 1.75, *p* = 0.191, nor interaction, *F*_(2,65)_ = 0.65, *p* = 0.526. To explore the main effect of age, we conducted post hoc mean comparisons between the age groups with Tukey HSD corrections for multiple comparisons. We identified that young adults showed lower scores on the MAAS compared with older adults, *p* < 0.001 (mean difference = 0.74, SE = 0.19, 95% CI [−1.20 −0.27]), but not compared with middle-aged adults, *p* = 0.122 (mean difference = 0.39, SE = 0.19, 95% CI [−0.86 0.08]). There was no difference between middle-aged and older adults, *p* = 0.222 (mean difference = 0.35, SE = 0.21, 95% CI [−0.85 0.15]). Over the audiobook period, we again identified a significant main effect of age, *F*_(2,65)_ = 7.93, *p* < 0.001, *η_p_*^2^ = 0.196, but no main effects of time, *F*_(1,65)_ = 0.03, *p* = 0.875, nor interaction, *F*_(2,65)_ = 0.89, *p* = 0.414.

For the FFMQ-15 scores, over the mindfulness meditation period, we identified a significant main effect of age, *F*_(2,65)_ = 4.73, *p* = 0.012, *η_p_*^2^ = 0.127, but no main effects of time, *F*_(1,65)_ = 1.63, *p* = 0.206, nor interaction, *F*_(2,65)_ = 0.36, *p* = 0.701. As with the MAAS, we identified that young adults showed lower scores on the FFMQ-15 compared with older adults, *p* = 0.010 (mean difference = 6.86, SE = 2.27, 95% CI [−12.32 −1.40]), but not compared with middle-aged adults, *p* = 0.163 (mean difference = 4.26, SE = 2.31, 95% CI [−9.79 1.28]). There was no difference between middle-aged and older adults, *p* = 0.538 (mean difference = 2.61, SE = 2.44, 95% CI [−8.47 3.25]). Over the audiobook period, we again identified a significant main effect of age, *F*_(2,65)_ = 4.07, *p* = 0.022, *η_p_*^2^ = 0.111, no main effect of time, *F*_(1,65)_ = 0.01, *p* = 0.928, but identified a significant interaction, *F*_(2,65)_ = 3.58, *p* = 0.034, *η_p_*^2^ = 0.099. The audiobook intervention marginally improved scores in young adults (pre: 51.3, post: 53.0), *t*_(26)_ = 1.78, *p* = 0.086), but not in middle-aged (pre: 54.8, post: 53.9) and older adults (pre: 56.0, post: 55.1), *t*s_(20)_ < 0.700, *p*s > 0.492. Table 2 contains all descriptive statistics with the FFMQ-15 scores reported divided into the subcategories.

**Table 2.** Mindfulness questionnaires

Measure	Intervention	Young adults		Middle-aged adults		Older adults	
		Pre	Post	Pre	Post	Pre	Post
MAAS	Mindfulness	3.69 (0.24)	3.74 (0.19)	4.23 (0.34)	4.14 (0.28)	4.29 (0.18)	4.54 (0.15)
	Audiobook	3.79 (0.12)	3.68 (0.14)	3.98 (0.27)	4.03 (0.27)	4.67 (0.24)	4.48 (0.23)
FFMQ: observation	Mindfulness	3.44 (0.13)	3.42 (0.13)	3.93 (0.21)	3.97 (0.19)	3.95 (0.14)	3.81 (0.15)
	Audiobook	3.51 (0.13)	3.62 (0.13)	3.90 (0.18)	3.90 (0.18)	3.78 (0.16)	3.76 (0.18)
FFMQ: description	Mindfulness	3.27 (0.21)	3.49 (0.14)	3.7 (0.21)	3.60 (0.19)	3.87 (0.19)	3.81 (0.15)
	Audiobook	3.15 (0.14)	3.28 (0.14)	3.72 (0.21)	3.75 (0.26)	3.90 (0.14)	3.84 (0.21)
FFMQ: awareness	Mindfulness	3.30 (0.15)	2.27 (0.13)	3.58 (0.21)	3.45 (0.23)	3.70 (0.15)	3.71 (0.16)
	Audiobook	3.27 (0.13)	3.26 (0.14)	3.48 (0.23)	3.57 (0.23)	3.70 (0.17)	3.67 (0.16)
FFMQ: non-judgmental	Mindfulness	3.54 (0.17)	3.53 (0.13)	3.68 (0.24)	3.85 (0.21)	4.08 (0.20)	4.22 (0.21)
	Audiobook	3.32 (0.16)	3.69 (0.17)	3.78 (0.18)	3.93 (0.22)	4.13 (0.21)	3.90 (0.22)
FFMQ: nonreactivity	Mindfulness	3.19 (0.11)	3.23 (0.13)	3.32 (0.18)	3.43 (0.23)	3.27 (0.22)	3.83 (0.17)
	Audiobook	3.28 (0.12)	3.28 (0.12)	3.60 (0.17)	3.42 (0.16)	3.70 (0.16)	3.29 (0.21)

We report the mean baseline and post-mindfulness intervention scores for the MAAS (total score of 6) and the FFMQ subcategories (total score of 5).

### Compliance

By partnering with the research division of the Headspace application team, we received the backend software usage for each participant. Although only required to complete one session per day, we found some participants completed more sessions than required. After completing the required Basics course, these participants explored other modes of guided mindfulness meditation. During the mindfulness intervention period, young adults completed an average of 25 sessions (SE = 2.46), middle-aged adults completed an average of 36 sessions (SE = 3.65), and older adults completed an average of 34 sessions (SE = 4.33). From our records, we received daily feedback forms of the audiobook completion. We identified young adults completed an average of 22 sessions (SE = 1.96) of audiobook listening, middle-aged adults completed an average of 27 sessions (SE = 1.45), and older adults completed an average of 28 sessions (SE = 1.18). The at-home compliance in this study is comparable with previous reports using the mobile Headspace application (Mahlo and Windsor, 2021).

To check whether days of mindfulness compliance on the Headspace application influenced our results, we conducted 3 (Age: young, middle-aged, older) × 2 (Time: pre, post) repeated-measures ANCOVA analyses with compliance as a covariate. With MAAS scores as the dependent measure, we identified a significant compliance × time interaction, *F*_(1,63)_ = 5.08, *p* = 0.028, *η_p_*^2^ = 0.075. Partial correlations with days of compliance and post-intervention MAAS scores while control for pre-intervention scores demonstrated a positive relationship, *r*_(65)_ = 0.37, *p* = 0.002, indicating more app-based practice led to higher MAAS scores post intervention. In contrast, the parallel ANCOVA with FFMQ scores as the dependent measure did not show a significant compliance × time interaction, *F*_(1,63)_ = 0.56, *p* = 0.459. In addition, we identified significant compliance × time interactions for the following oculomotor measures in the singleton search task: first fixation to the distractor, *F*_(1,63)_ = 6.25, *p* = 0.015, *η_p_*^2^ = 0.090, first fixation to the target, *F*_(1,63)_ = 5.27, *p* = 0.025, *η_p_*^2^ = 0.077, and saccadic reaction times in distractor-present trials, *F*_(1,63)_ = 5.11, *p* = 0.027, *η_p_*^2^ = 0.075. Partial correlations with days of compliance and post-intervention measures while controlling for pre-intervention measures showed that more days of mindfulness practice led to more first fixations to the distractor, *r*_(65)_ = 0.27, *p* = 0.025, less first fixations to the target, *r*_(65)_ = −0.25, *p* = 0.038, but no significant relationship with saccadic reaction times, *r*_(65)_ = −0.23, *p* = 0.067. Interestingly, these findings showing that increased use of the Headspace application was associated with greater distractibility by the salient, task-irrelevant distractor and less goal-directed attentional control were contrary to our predictions.

## Discussion

This is the first study to show evidence that guided mindfulness meditation via a mobile application improves some mechanisms of attentional control using eye tracking. We used two oculomotor search tasks that manipulated a salient color distractor which is typically suppressed (feature search task) or disengaged from after fixation (singleton search task), given the high reliability of these attention-related eye movement measures in prior studies (internal consistency; Kim et al., 2024a) and in our results (test–retest reliability). By using daily email reminders and requiring participants to complete a short feedback form after each daily intervention session, we maintained high at-home compliance from our participants during the study. Although older adults showed higher mindfulness scores on the two mindfulness questionnaires compared with young adults across all visits, we identified that the effect of mindfulness practice is only observable in oculomotor measures, which may be more sensitive to longitudinal changes compared with self-report questionnaires.

Prior studies investigating the beneficial effects of mindfulness meditation on attention have primarily investigated Posner and Petersen's (1990) model of attentional networks using the Attention Network Task or as a mode of executive control (see Sumantry and Stewart, 2021, for a meta-analysis). In the Attention Network Task, mindfulness training has previously shown to improve modes of orienting and executive control but not alerting (Ainsworth et al., 2013; Becerra et al., 2017) and also improve overall reaction times (Mora Álvarez et al., 2023). Furthermore, in a Go/NoGo Task, mindfulness training showed differences on its effect on proactive and reactive mechanisms of attentional control as in our study with improvements in reactive mechanisms but not in proactive control (Fountain-Zaragoza et al., 2016). Interestingly, we found one study using a visual search task which showed that mindfulness exhibited a null effect on task performance (Wimmer et al., 2020). These findings suggest that mindfulness does not broadly improve all mechanisms of attentional control, but effects depend on the neural networks regulating those attention processes. One neuroimaging study has identified that focused breathing and mindful attention increases brain activity in the dorsomedial prefrontal cortex, temporoparietal junction, and the dorsal anterior cingulate cortex (Dickenson et al., 2013). These brain regions are also activated in both visual search tasks used in this study and control attention orienting, reorienting, and switching between the ventral and dorsal attention streams (Corbetta and Shulman, 2002; Corbetta et al., 2008). Given that we did not identify age differences in the effects of mindfulness meditation in our study, the global benefits of mindfulness meditation across all participants suggest that the effects of mindfulness on attention may be achieved through these core attention networks.

Across multiple analyses, we consistently observed that guided mindfulness meditation practice improved mean saccadic reaction times. Saccadic reaction times, or the time to initiate the first saccade following the onset of the stimulus array, have been commonly used to measure cognitive function (Zhang et al., 2025), including in the field of aging and neurodegenerative diseases (Campbell and Ryan, 2009; Molitor et al., 2015; Chehrehnegar et al., 2019; Koçoğlu et al., 2021; Herrmann and Ryan, 2024; Qi et al., 2024). The neural correlates of this measure have shown to be initiated in the visual striate cortex and controlled through the superior colliculus and frontal eye fields (Maunsell and Gibson, 1992; Dorris and Munoz, 1995; Dorris et al., 2002). These neural networks, in the context of visual spatial attention mechanisms, coordinate and plan eye movements prior to initiation (Krauzlis et al., 2013). In macaque monkeys, the administration of a selective noradrenaline reuptake inhibitor have shown that the latencies of these early impulsive saccades are mediated by the locus ceruleus-noradrenaline system (Suzuki and Tanaka, 2017) and that faster responses may be because of increased spatial perceptivity to salient stimuli (Ghosh and Maunsell, 2024). Even though participants were successful in proactively inhibiting the salient distractor in this task across all trials, evidence of faster saccadic reaction times to the distractor suggests that mindfulness practice may have modulated the cognitive perception of the salient but task-irrelevant stimulus that ultimately influenced the speed of attention processing. However, from our findings alone, it is unclear whether this effect of mindfulness on attention is specifically because of a mediating effect on the LC-NA system. Furthermore, given this significant effect on saccadic latencies, it is also plausible that the other mechanisms of attentional control are just not sensitive to these changes (e.g., dwell time, fixation time, first fixations), as multiple other neuromodulatory systems are involved in modulating the direction of eye movements and also in reorienting and disengagement.

However, an alternative interpretation is that mindfulness practice, by enhancing present-moment awareness, may increase attention bias toward salient stimuli even when they are task irrelevant. Although mindfulness is thought to decrease distractibility by increasing general focus, a previous randomized controlled trial has also shown that the practice of being receptive to present-moment experiences may enhance awareness of the environment which may at times be detrimental to the task at hand (Semple, 2010). Specifically, in the oculomotor search paradigms used here, this increased awareness would have increased the attentional priority of the salient stimulus, leading to greater distractibility by a task-irrelevant shape. Interestingly, we only identified increased saccadic reaction times in the feature search task in which the salient distractor is proactively inhibited and not in the singleton search task in which the distractor is reactively rejected. Cognitive control of automatic inhibitory processes can be both proactive and reactive (Ballanger, 2009; Aron, 2011) and involve different neural networks (Talanow et al., 2020). Our findings suggest that practicing mindfulness influences inhibitory mechanisms that proactively prevent inappropriate responses, such as saccadic eye movements, that is controlled in a neural network involving the frontal eye fields, dorsolateral prefrontal cortex, and intraparietal sulcus (Anderson et al., 2008). These neural correlates have indeed been previously implicated to be modulated by mindfulness meditation in a previous fMRI study (Dickenson et al., 2013). It is plausible that increased awareness of the present moment that results from mindfulness practice may lead to a top-down modulation of visual attentional priority that is occurring prestimulus visualization. Our findings show that the effects of mindfulness may not be simple changes to attention processing, such as increased vigilance or heightened alertness that would be evident with changes in the direction of eye movements in our tasks. Rather, these collective findings suggest that mindfulness may alter the interactions between the top-down influence of attentional control on the attentional priority maps within the visual cortex, leading to specific changes that may or may not be beneficial depending on the task requirements. Future research investigating the changes between these mechanisms of attentional control among expert mindfulness meditators may clarify these unanswered hypotheses.

In addition, we identified longitudinal improvements in measures for goal-directed attentional control and distractibility by task-irrelevant stimuli. However, most of these improvements were observable following both the mindfulness meditation and the positive control audiobook interventions. Furthermore, our post hoc analyses suggest these improvements may be attributed to practice effects and/or potentially completing a daily relaxing task. Prior studies have shown that performance in these visual search tasks rapidly improves in early trials from practice but quickly reaches maximal performance (Kim et al., 2024b). Furthermore, the beneficial effects of prior experience in similar visual search tasks are well characterized, and learned associations have shown to persist months after the initial experiment (Anderson and Yantis, 2013; Anderson et al., 2021). These findings suggest that the attention system can become better facilitated to orient eye movements toward targets in the presence of a salient, task-irrelevant distractor. The practice effects in this task may have been stronger given that the feature-specific nature of the distractor was consistent in appearance throughout the experiment (Kelley and Yantis, 2009). Our results emphasize the importance of utilizing a positive control (e.g., audiobook) and examining for practice effects when investigating the longitudinal benefits of mindfulness meditation practice on measures of cognition.

In a preregistered study, we demonstrate that guided mindfulness meditation via the Headspace application significantly enhances attentional control mechanisms, as measured through eye movements. These findings highlight two key insights. First, they support the effectiveness of mobile-based mindfulness meditation as a tool for improving cognitive function. Second, they build upon previous research suggesting that short-term mindfulness training (i.e., 30 d) is sufficient to influence neural processing mechanisms of attention (Tang et al., 2007; Zeidan et al., 2010; Howarth et al., 2019; Mahlo and Windsor, 2021; Gavrilova and Zawadzki, 2023). Notably, we did not find evidence that older adults benefitted more from the mindfulness meditation intervention than young adults, suggesting the mindfulness meditation may be equally beneficial across all age groups. While we identified an improvement in one measure of attention mediated by the LC-NA system, it remains unclear whether mindfulness training specifically modulates this neuromodulatory system over time. Additionally, self-report data from the MAAS and FFMQ questionnaires revealed that younger adults scored lower than older adults. However, unlike Champion et al. (2018), we found no intervention-related effects on these mindfulness scores, suggesting that cognitive measures derived from eye tracking may be more sensitive indicators of change than self-report assessments. Notably, it remains plausible that app-based guided mindfulness practices exert weaker effects on these outcome measures compared with in-person group training. Meta-analytic reviews have highlighted the limited efficacy of smartphone-based mindfulness interventions, often reporting small effect sizes (Fish et al., 2016; Wu et al., 2022). Nevertheless, randomized controlled trials with independent mobile app and in-person mindfulness-based program groups have showed that both modes of mindfulness training are equally effective in reducing anxiety (Orosa-Duarte et al., 2021), while another showed both modalities are effective in decreasing stress but only the in-person training improved life satisfaction (Hoover et al., 2022). These findings lay the groundwork for future longitudinal studies exploring the effects of mindfulness on cognitive processes and highlight critical methodological considerations for such research.

## Synthesis

Reviewing Editor: Ifat Levy, Yale School of Medicine Decisions are customarily a result of the Reviewing Editor and the peer reviewers coming together and discussing their recommendations until a consensus is reached. When revisions are invited, a fact-based synthesis statement explaining their decision and outlining what is needed to prepare a revision will be listed below. The following reviewer(s) agreed to reveal their identity: NONE.

The authors have successfully carried out the pre-registered study - it is well-executed, the results are interesting and novel, and the paper is well written. There are minor comments that the authors should address before acceptance:

1. Results: The authors mention that some individuals practiced more sessions than was required per day and report group averages of the number of sessions. Was this difference in number of sessions controlled for in the ANOVAs and other statistical analyses?

2. Discussion: It's interesting that the self-report mindfulness scores did not significantly change after the mindfulness intervention. The authors raised some potential speculations as to why this may be the case. However, it would be useful to discuss the possibility that the modality of how the intervention was administered could potentially contribute to the difference between the present findings and those of in-person group interventions that observed changes in self-report mindfulness. It is likely that the eye tracking measure is more sensitive, but we cannot completely rule out that in-person group training may have stronger effects on these measures compared to app-based guided practices.

3. The authors are strongly encouraged to add more information about the results distribution beyond bar graph (e.g. violin graphs, or plotting individual points on top of the bars).
