# Supplementary material for: The Effects of Mindfulness Meditation on Mechanisms of Attentional Control in Young and Older Adults: A Preregistered Eye Tracking Study
Source: eNeuro. 2025 Jul 22;12(7):ENEURO.0356-23.2025. doi: 10.1523/ENEURO.0356-23.2025 (PMC12301956; doi:10.1523/ENEURO.0356-23.2025)
Supplement: Figure 2-1 — Extended data table supporting Figure 2 with descriptive statistics. All values are presented as means and standard errors in parentheses. Download Figure 2-1, DOCX file. [file eneuro-12-ENEURO.0356-23.2025-s001.docx]

|  |  |  | **Young Adults** | | **Middle-Aged Adults** | | **Older Adults** | |
| --- | --- | --- | --- | --- | --- | --- | --- | --- |
| **Task** | **Measure** | **Intervention** | **Pre** | **Post** | **Pre** | **Post** | **Pre** | **Post** |
| Feature Search  Task | First Saccade to Distractor (%) | Mindfulness | 4.62 (0.81) | 3.71 (0.71) | 8.59 (1.75) | 8.22 (1.84) | 9.96 (1.81) | 7.42 (1.25) |
|  |  | Audiobook | 3.85 (0.54) | 2.87 (0.48) | 9.48 (1.95) | 6.63 (0.99) | 8.87 (1.32) | 8.39 (1.43) |
|  | Oculomotor Suppression (%) | Mindfulness | 1.35 (0.96) | 1.27 (0.68) | 2.29 (2.04) | 1.23 (1.80) | 1.95 (1.97) | 3.64 (1.36) |
|  |  | Audiobook | 1.93 (0.70) | 2.57 (0.82) | 0.60 (1.89) | 3.14 (1.34) | 3.77 (1.49) | 2.55 (1.67) |
| Singleton Search  Task | First Saccade to Distractor (%) | Mindfulness | 18.64 (2.01) | 16.77 (2.05) | 25.83 (3.12) | 22.89 (2.78) | 30.90 (2.76) | 29.77 (2.60) |
|  |  | Audiobook | 19.20 (2.07) | 17.87 (2.00) | 24.97 (2.77) | 24.36 (3.63) | 31.98 (2.69) | 31.64 (2.98) |
|  | Oculomotor Capture (%) | Mindfulness | 12.35 (2.19) | 10.62 (2.25) | 15.69 (3.29) | 13.00 (2.92) | 18.77 (3.23) | 18.56 (3.09) |
|  |  | Audiobook | 13.38 (2.24) | 11.50 (2.17) | 15.17 (2.69) | 15.87 (3.86) | 19.11 (3.12) | 20.13 (3.37) |

**Figure 2-1.** Extended data table supporting Figure 2 with descriptive statistics. All values are presented as means and standard errors in parentheses.
