# Supplementary material for: The Effects of Mindfulness Meditation on Mechanisms of Attentional Control in Young and Older Adults: A Preregistered Eye Tracking Study
Source: eNeuro. 2025 Jul 22;12(7):ENEURO.0356-23.2025. doi: 10.1523/ENEURO.0356-23.2025 (PMC12301956; doi:10.1523/ENEURO.0356-23.2025)
Supplement: Figure 3-1 — Extended data table supporting Figure 3 with descriptive statistics. All values are presented as means and standard errors in parentheses. Download Figure 3-1, DOCX file. [file eneuro-12-ENEURO.0356-23.2025-s002.docx]

|  |  |  | **Young Adults** | | **Middle-Aged Adults** | | **Older Adults** | |
| --- | --- | --- | --- | --- | --- | --- | --- | --- |
| **Task** | **Measure** | **Intervention** | **Pre** | **Post** | **Pre** | **Post** | **Pre** | **Post** |
| Feature Search  Task | First Saccade to Target in Distractor Absent Trials (%) | Mindfulness | 82.58 (2.28) | 87.11 (1.86) | 70.21 (2.97) | 72.39 (3.81) | 67.66 (3.98) | 72.84 (3.49) |
|  |  | Audiobook | 84.30 (1.96) | 86.30 (2.30) | 72.00 (3.80) | 74.29 (3.77) | 69.39 (3.51) | 70.59 (3.44) |
|  | First Saccade to Target in Distractor Present Trials (%) | Mindfulness | 83.43 (1.91) | 86.33 (1.78) | 69.65 (2.93) | 72.88 (3.32) | 66.22 (3.63) | 70.46 (3.13) |
|  |  | Audiobook | 84.59 (1.77) | 86.25 (1.72) | 70.37 (3.34) | 73.84 (3.14) | 65.85 (3.32) | 69.74 (3.14) |
| Singleton Search  Task | First Saccade to Target in Distractor Absent Trials (%) | Mindfulness | 83.37 (1.55) | 83.24 (1.93) | 73.16 (2.39) | 74.88 (2.99) | 63.72 (2.93) | 65.37 (2.79) |
|  |  | Audiobook | 82.48 (1.48) | 81.82 (1.66) | 74.26 (2.81) | 76.52 (2.44) | 59.60 (2.85) | 63.94 (3.18) |
|  | First Saccade to Target in Distractor Present Trials (%) | Mindfulness | 68.79 (2.16) | 70.93 (2.21) | 53.89 (3.44) | 57.32 (3.47) | 44.83 (2.63) | 47.81 (2.71) |
|  |  | Audiobook | 69.17 (2.10) | 69.340 (2.10) | 55.44 (3.72) | 58.65 (3.95) | 42.28 (3.00) | 45.33 (3.16) |

**Figure 3-1.** Extended data table supporting Figure 3 with descriptive statistics. All values are presented as means and standard errors in parentheses.
