# Supplementary material for: The Effects of Mindfulness Meditation on Mechanisms of Attentional Control in Young and Older Adults: A Preregistered Eye Tracking Study
Source: eNeuro. 2025 Jul 22;12(7):ENEURO.0356-23.2025. doi: 10.1523/ENEURO.0356-23.2025 (PMC12301956; doi:10.1523/ENEURO.0356-23.2025)
Supplement: Figure 4-1 — Extended data table supporting Figure 4 with descriptive statistics. All values are presented as means and standard errors in parentheses. Download Figure 4-1, DOCX file. [file eneuro-12-ENEURO.0356-23.2025-s003.docx]

|  |  |  | **Young Adults** | | **Middle-Aged Adults** | | **Older Adults** | |
| --- | --- | --- | --- | --- | --- | --- | --- | --- |
| **Task** | **Measure** | **Intervention** | **Pre** | **Post** | **Pre** | **Post** | **Pre** | **Post** |
| Feature Search  Task | Fixation Time (ms) | Mindfulness | 468.92 (13.68) | 436.77 (9.28) | 510.77 (16.87) | 515.05 (20.38) | 556.95 (24.92) | 551.66 (26.24) |
|  |  | Audiobook | 438.98 (8.96) | 435.95 (10.83) | 520.89 (22.56) | 494.22 (16.35) | 567.77 (27.45) | 551.70 (22.99) |
|  | Saccadic RT (ms) | Mindfulness | 228.28 (11.52) | 198.97 (14.63) | 258.23 (16.97) | 220.78 (11.09) | 252.78 (14.03) | 248.89 (25.75) |
|  |  | Audiobook | 212.51 (12.54) | 224.80 (9.99) | 213.27 (10.62) | 240.22 (12.93) | 253.30 (24.87) | 247.19 (13.32) |
|  | Dwell Time (ms) | Mindfulness | 186.22 (14.61) | 148.34 (9.43) | 177.23 (15.88) | 171.62 (11.37) | 167.73 (11.54) | 186.02 (9.22) |
|  |  | Audiobook | 164.22 (12.02) | 180.67 (21.08) | 164.00 (9.22) | 188.34 (19.02) | 178.85 (8.15) | 172.90 (11.24) |
| Singleton Search  Task | Fixation Time (ms) | Mindfulness | 573.26 (15.25) | 536.17 (14.71) | 709.18 (30.38) | 689.85 (25.88) | 755.85 (28.22) | 755.56 (37.21) |
|  |  | Audiobook | 552.97 (11.99) | 521.97 (9.97) | 715.25 (30.67) | 668.64 (28.16) | 771.83 (37.36) | 720.60 (28.66) |
|  | Saccadic RT (ms) | Mindfulness | 273.39 (4.98) | 265.90 (8.18) | 283.88 (11.25) | 289.45 (13.60) | 293.32 (9.89) | 294.02 (8.63) |
|  |  | Audiobook | 284.47 (8.22) | 260.18 (3.78) | 293.95 (14.13) | 281.69 (11.10) | 291.39 (9.10) | 293.53 (8.30) |
|  | Dwell Time (ms) | Mindfulness | 186.21 (7.72) | 168.67 (5.13) | 232.30 (9.23) | 251.69 (15.03) | 229.39 (11.12) | 240.36 (15.34) |
|  |  | Audiobook | 178.20 (5.43) | 173.40 (7.36) | 251.62 (14.95) | 232.11 (10.25) | 252.39 (16.86) | 229.43 (11.26) |

**Figure 4-1.** Extended data table supporting Figure 4 with descriptive statistics. All values are presented as means and standard errors in parentheses.
